# Clinicopathological Features, Tumor Mutational Burden, and Tumour-Infiltrating Lymphocyte Interplay in *ERBB2*-Mutated Breast Cancer: *In Silico* Analysis

**DOI:** 10.3389/pore.2021.633243

**Published:** 2021-04-01

**Authors:** Shiro Uchida, Takaaki Kojima, Takashi Sugino

**Affiliations:** ^1^ Division of Diagnostic Pathology, Kikuna Memorial Hospital, Yokohama, Japan; ^2^ Division of Pathology, Shizuoka Cancer Center, Shizuoka, Japan; ^3^ Department of Human Pathology, Juntendo University School of Medicine, Tokyo, Japan; ^4^ Graduate School of Bioagricultural Sciences, Nagoya University, Nagoya, Japan

**Keywords:** Breast cancer, ErbB2 mutation, tumor mutational burden, tumour-infiltrating lymphocytes, Bioinformactics analysis

## Abstract

Recent evidence suggests that somatic mutations in *ERBB2* activate *ERBB2* signaling. These mutations occur at a frequency of approximately 3% in breast cancer (BC). *ERBB2* mutations indicate poor prognosis as they are associated with recurrence and metastasis. This study aimed to evaluate the clinicopathological features, immune infiltration levels, tumor mutational burden (TMB), and tumor-infiltrating lymphocytes (TILs) in *ERBB2*-mutated breast cancer (*ERBB*2-mutated BC) using a bioinformatic approach and publicly available datasets (i.e., TCGA-BRCA and TIMER2.0). *ERBB2*-mutated BCs were associated with a high histological grade. *ERBB2*-mutated BCs comprised invasive breast carcinoma of no special type (21/35, 60%), classic invasive lobular carcinoma (12/35, 34.3%), and pleomorphic invasive lobular carcinoma (2/35, 5.7%). A Kaplan-Meier survival curve demonstrated that *ERBB2*-mutated BC was associated with a significantly worse prognosis compared to *ERBB2* non-mutated BC (*p* < 0.01). Furthermore, 40% (14/35) of the patients with *ERBB2*-mutated BC harbored *CDH1* mutations. Mutations at L755 and V777 accounted for 30.5% of these mutations in *ERBB2*-mutated BC, suggesting that these sites are mutational hot spots in BC, particularly in invasive lobular carcinoma. Of the *ERBB2*-mutated BCs, 8.6% were classified as TIL-high, whereas 77.1% were TILs-low; TMB significantly correlated with TILs (*p* < 0.05). CD8^+^ T cell infiltration levels were significantly higher in *ERBB2* non-mutated BC. Among *ERBB2*-mutated BCs, 22.9% were classified as TMB-high, which was significantly higher than the rate in the *ERBB2* non-mutated BC (*p* < 0.01). These findings provide evidence for a link between *ERBB2* mutations and high TMB in BC.

## Introduction

It has been recently reported that in rare cases, somatic mutations in *ERBB2* can activate *ERBB2* signaling [1]. *In vitro* analyses have demonstrated that some *ERBB2* mutations are oncogenic and promote cancer cell growth, invasion, and survival [1, 2]. A systematic review revealed that the frequency of *ERBB2* mutations in breast cancer (BC) is relatively low (2.7%) [3]. *ERBB2* mutations are more common in invasive lobular carcinoma (ILC) than in invasive breast carcinoma of no special type (IBC-NST) [4]. ILC is classified into two subtypes, classic ILC (c-ILC) and pleomorphic ILC (*p*-ILC). *CDH1* is located on chromosome 16q22.1 and encodes E-cadherin [5], a transmembrane glycoprotein expressed in epithelial tissues and contributes to calcium-dependent cell-to-cell adhesion [6]. Patients with relapsed *CDH1*-mutated ILC exhibit a higher frequency of *ERBB2* somatic mutations than those with non-*CDH1*-mutated BC [7]. *ERBB2* mutations indicate a significantly poor prognosis, regardless of the histological type [8, 9] as they are associated with relapse and bone metastasis [7, 10]. Previous studies have demonstrated that the *ERBB2* mutation is frequently associated with ILC [7, 10–14]. The *CDH1* mutation is a major gene mutation in ILC, but only a few studies have focused on histopathological images and mutations of *ERBB2* and *CDH1* [12, 14]. Moreover, the number of *ERBB2*-mutated BC was relatively fewer, 5 cases and 18 cases, respectively. Therefore, the relationship between the histological type and genetic alteration of *ERBB2* and *CDH1* has not been completely elucidated. In this study, we investigated the clinicopathological characteristics and the frequency of co-occurrence of the *CDH1* mutation and tumour-infiltrating lymphocytes (TILs) in 35 *ERBB2*-mutated BCs via a public database (TCGA-BRCA). Furthermore, we compared the tumor mutational burden (TMB) as well as CD8^+^ T cell, CD4^+^ T cell, and Treg immune infiltration levels between *ERBB2*-mutated BC and *ERBB2* non-mutated BC via *in silico* analysis.

## Materials and Methods

### Data Collection

Data on *ERBB2* and *CDH1* somatic mutations were obtained from UCSC Xena (http://xena.ucsc.edu/). Genomic Data Commons (GDC) TCGA Breast *Cancer* (BRCA), comprising 986 BC samples, was used to obtain mutation data. Clinicopathological information (age of onset, ethnicity, sex, histology, and subtype) and genomic information from TCGA-BRCA were obtained using the GDC Data Portal (https://portal.gdc.cancer.gov) (Supplementary Table S1). The *ERBB2* mutation type, amino acid change, and mutation site were identified. The *ERBB2* mutation site was classified as follows: Receptor-L domain, Furin-like cysteine-rich domain, kinase domain, interdomain region, or C-terminal region. The pathological significance of each *ERBB2* or *CDH1* mutation was assessed using COSMIC FATHMM, Ensembl Variant Effect Predictor (VEP), SHIFT, and PolyPhen (Supplementary Table S2). Statistical analyses were performed using R software, version 4.0.3.

### Clinicopathological Features

Clinicopathological features, including age, sex, ethnicity, and intrinsic subtype data were obtained from the GDC Data Portal. Intrinsic subtypes, as determined using multi-gene assay PAM 50, were classified into the following five types: Luminal A (LumA), Luminal B (LumB), Her-2, Basal, and normal. However, there were cases in which subtypes were not assigned and others with two subtypes; these were excluded. Finally, there were 32 cases in *ERBB2*-mutated BC and 859 cases in *ERBB2* non-mutated BC. The prognosis of *ERBB2* mutated BC was compared with that of *ERBB2* non-mutated BC and analyzed on the Kaplan–Meier survival curve using UCSC Xena (http://xena.ucsc.edu/).

### Pathological Review

**FIGURE 1:**
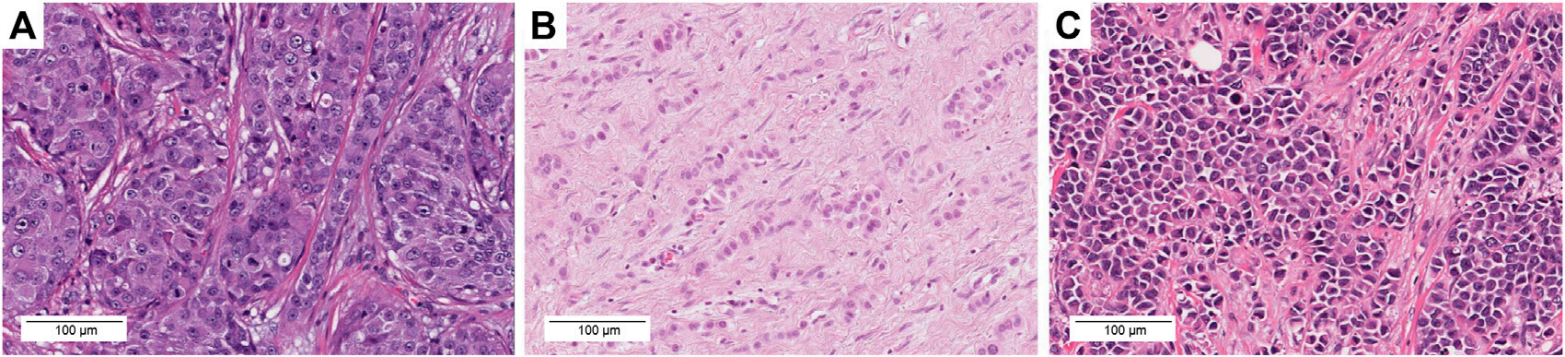
Representative images of each histological type with histological grade, molecular subtype, *ERBB2* mutation, *CDH1* mutation, TMB, and TILs. **(A)** TCGA-A2-A3XV-01A. H&E images, IBC-NST, GII, Her2 type. The tumor exhibited a solid growth pattern and was composed of tumor cells with severe nuclear atypia. S310F *ERBB2* mutation, TMB = 1.8 mut/Mb, TILs = 10%. **(B)** TCGA-D8-A27G-01A. H&E images, c-ILC, GII, LumA type. This tumor exhibited a linear growth pattern and loose cohesion; tumor cell proliferation was observed along with mild nuclear atypia. I767M *ERBB2* mutation, G169Rfs*5 *CDH1* mutation, TMB = 31.3 mut/Mb, TILs = 0%. **(C)** TCGA-BH-A18P-01A. H&E images, *p*-ILC, GIII, Her2 type. This tumor exhibited a solid growth pattern and loose cohesion; tumor cell proliferation was observed along with severe nuclear atypia. L755S *ERBB2* mutation, S36Afs*20 *CDH1* mutation, TMB = 11.8 mut/Mb, TILs = 10%. Scale bar = 100 μm, ×20 magnification.

Samples with *ERBB2* mutations (*n* = 35) were reassessed. Whole slide images of diagnostic sections in *ERBB2*-mutated cases (*n* = 35) were downloaded from the GDC Data Portal and analyzed using Aperio ImageScope (Sausalito, CA, United States). The histological types were classified according to the 5th edition of the World Health Organization classification system [15]. The histological grade was evaluated in accordance with the Elston and Ellis modification of the Nottingham grading system [16]. All samples were reviewed by two pathologists (S.U. and T.S.). Interobserver differences were resolved through re-evaluation and discussion to reach consensus. Samples were defined as c-ILC when all tumor cells were small and uniform with round nuclei and inconspicuous nucleoli (Figure 1B). *p*-ILC was defined based on previously reported cytological characteristics (i.e., a greater degree of cellular pleomorphism, nuclear membrane irregularities, more prominent nucleoli, increased hyperchromasia, and more frequent mitotic activity) (Figure 1C) [17].

### Assessment of TILs

Stromal TILs were quantified on each virtual slide. TILs were assessed in accordance with the guidelines proposed by the international TILs working group [18]. Stromal TILs counts were estimated as the percentage of immune cells in stromal tissues within the tumor. TILs were categorized into three: low (0–10%), intermediate (11–59%), and high (60–100%).

### Timer2.0 Database Analysis

To compare *ERBB2*-mutated BC and *ERBB2* non-mutated BC for immune infiltration levels of immune cells including CD8^+^ T cell, CD4^+^ T cell, and Treg, the online public resource, Tumor Immune Estimation Resource 2.0 (TIMER2.0; https://timer.cistrome.org/), was utilized [19].

### TMB Estimation

TMB is a measure of the total number of mutations per megabase of tumor tissue. It can also be interpreted as the mutation density in tumor genes, defined as the average number of mutations in the tumor genome, including the total number of coding sequence errors, base substitutions, insertion, or deletions [20]. TMB was estimated for TCGA-BRCA (*n* = 951) and *ERBB2*-mutated BC (*n* = 35) and was calculated as the total number of mutations per sample/38, with an estimated exome size of 38 Mb [21]. The samples were classified as TMB-high if they had ≥10 mutations per megabase (mut/Mb) as previously described [22].

### Statistical Analysis

The subtype, CDH1 mutation, and TMB between *ERBB2* mutated BC and *ERBB2* non-mutated BC were analyzed by Fisher exact tests, with a significance threshold of *p* < 0.05. The correlations for each TMB and TILs were evaluated using the Spearman rank correlation coefficient. Results with *p* < 0.05 were considered statistically significant.

## Results

### Clinicopathological Properties for BC With *ERBB2* Mutations

**FIGURE 2:**
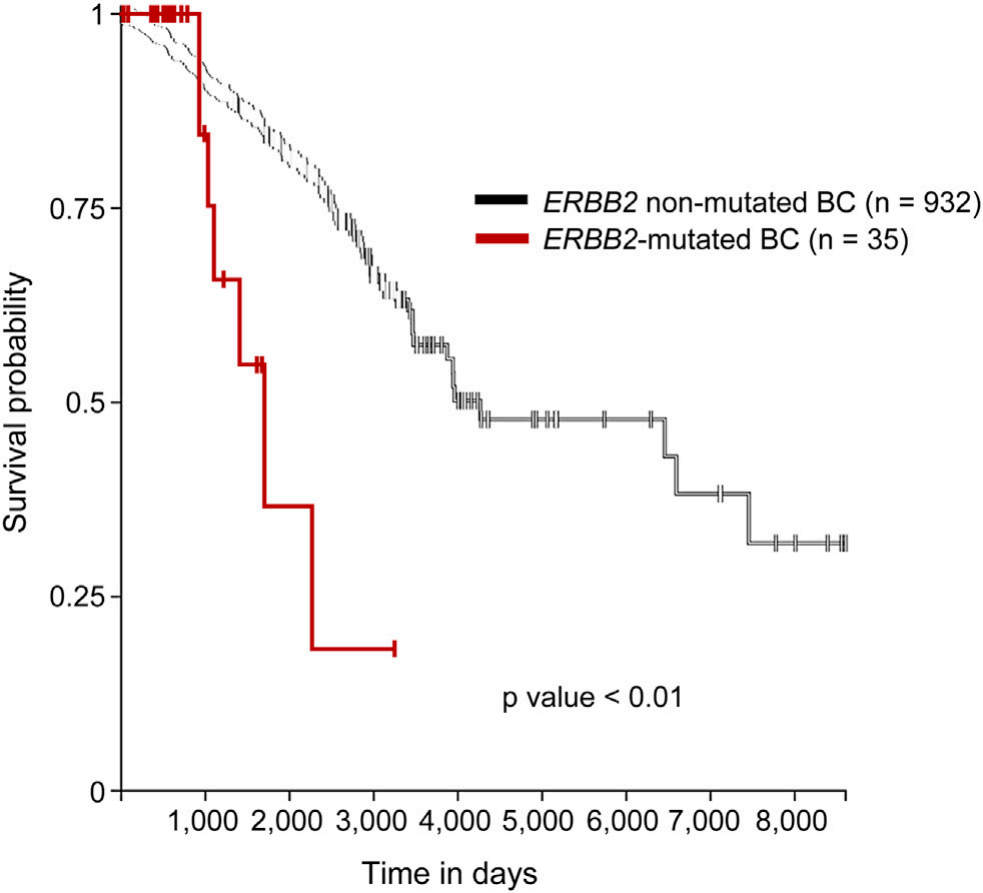
Survival analysis of the mutated and non-mutated *ERBB2* BC cases. Kaplan–Meier survival curve demonstrated that *ERBB2*-mutated BC had a significantly worse prognosis than did *ERBB2* non-mutated BC. Purple line; *ERBB2* mutated BC, black line; *ERBB2* non-mutated BC.

We identified 35 samples of *ERBB2*-mutated BC among 986 BC samples in TCGA-BRCA (35/986, 3.5%). Two samples (TCGA-A2-A0T6-01A and TCGA-C8-A3M7-01A) exhibited three distinct *ERBB2* mutations. Therefore, a total of 39 mutations were identified in 35 samples. The histological types, subtypes, variant types, amino acid changes, mutation sites, TMB, TILs, and *CDH1* mutations for each sample are summarized in Supplementary Table S3. The clinicopathological characteristics and TILs for *ERBB2*-mutated BC are summarized in Table 1. The comparison of ERBB2-mutated BC and ERBB2 non-mutated BC, with respect to subtype, co-occurrence of *CDH1* mutation, and TMB, is summarized in Table 2. Briefly, 34 women and 1 man were affected with *ERBB2*-mutated BC (mean age, 61.7 years; range, 31–88 years). The 35 breast samples corresponded to 21 IBC-NST (60%) cases, 12 c-ILC (34.3%) cases, and two *p*-ILC (5.7%) cases (Table 2). *ERBB2* somatic mutations were detected in 35 samples, and the co-occurrence of *ERBB2* and *CDH1* mutations was observed in 14 samples (Supplementary Tables S4, S5). *CDH1* mutations were observed at significantly higher frequencies (40%; 14/35) in patients with *ERBB2* mutated BC than in those with *ERBB2* non-mutated BC (Table 2). *ERBB2*-mutated BCs showed a high histological grade (including a predominantly solid growth pattern, rarely tubule formation, remarkable nuclear atypia, and high mitotic counts) (Figure 1A). The subtypes LumA, LumB, HER2, basal, and normal were detected in 56.3% (18/32), 12.5% (4/32), 18.8% (6/32), 3.1% (1/32), and 9.4% (3/32) of patients, respectively. *ERBB2* mutations were present in all subtypes, especially in LumA. However, the subtype proportion between *ERBB2* mutated BC and *ERBB2* non-mutated BC was not significant. *ERBB2*-mutated BC had a significantly worse prognosis than *ERBB2* non-mutated BC (*p* < 0.01) (Figure 2).

**TABLE 1:** Clinicopathological information for *ERBB2*-mutated BC in the TCGA-BRCA cohort.

Categories		*ERBB2*-mutated BC (*n* = 35)	
Age of onset	Mean (range)	61.7 years (31–88 years)	
		Patients	%
Sex	Male	1	2.9
	Female	34	97.1
Ethnicity	Caucasian	20	57.1
	African or African American	5	14.3
	Asian	3	8.6
	Not reported	7	20
Histology	IBC-NST	21	60
	c-ILC	12	34.3
	*p*-ILC	2	5.7
Histological grade	I	2	5.7
	II	21	60
	III	12	34.3
TILs (%)	Low (0–10%)	27	77.1
	Intermediate (11–59%)	5	14.3
	High (60–100%)	3	8.6

*ERBB2*-mutated BC: *ERBB2*-mutated breast cancer; IBC-NST: Invasive breast cancer-no special type; c-ILC: Classic invasive lobular carcinoma; *p*-ILC: Pleomorphic invasive lobular carcinoma; TILs: Tumour-infiltrating lymphocytes; TMB: Tumor mutational burden.

**TABLE 2:** Comparison of *ERBB2*-mutated BC and *ERBB2* non-mutated BC for subtype and co-occurrence of the *CDH1* mutation and TMB.

Categories		*ERBB2*-mutated BC		*ERBB2* non-mutated BC		*p* value
		Patients	%	Patients	%	
Subtype	Lum A	18	56.3	432	50.4	0.59
	Lum B	4	12.5	177	20.7	0.37
	Her2	6	18.8	154	18.0	0.82
	Basal	1	3.1	64	7.5	0.72
	Normal	3	9.4	30	3.5	0.11
*CDH1* mutation	Present	14	40	127	13.3	<0.01
	Absent	21	60	824	86.6	
TMB	Low (<10/Mb)	27	77.1	922	97	
	High (≧10/Mb)	8	22.9	29	3	<0.01

*ERBB2*-mutated BC: *ERBB2*-mutated breast cancer; *ERBB2* non-mutated BC: *ERBB2* non-mutated breast cancer; Lum A: Luminal A; Lum B: Luminal B; TMB: Tumor mutational burden.

### Mutation Type and Distribution of *ERBB2* Mutation Sites

Of the 39 mutations, 29 were missense mutations (74.4%), four were synonymous mutations (10.2%), two were in-frame insertions (5.1%), one was an in-frame deletion (2.5%), two were present in intronic regions (5.1%) (chr17:g.39712114C > A, chr17:g.39712166C > G), and one was present in the 3′-UTR (2.5%) (chr17:g.39729470G > A).

**FIGURE 3:**
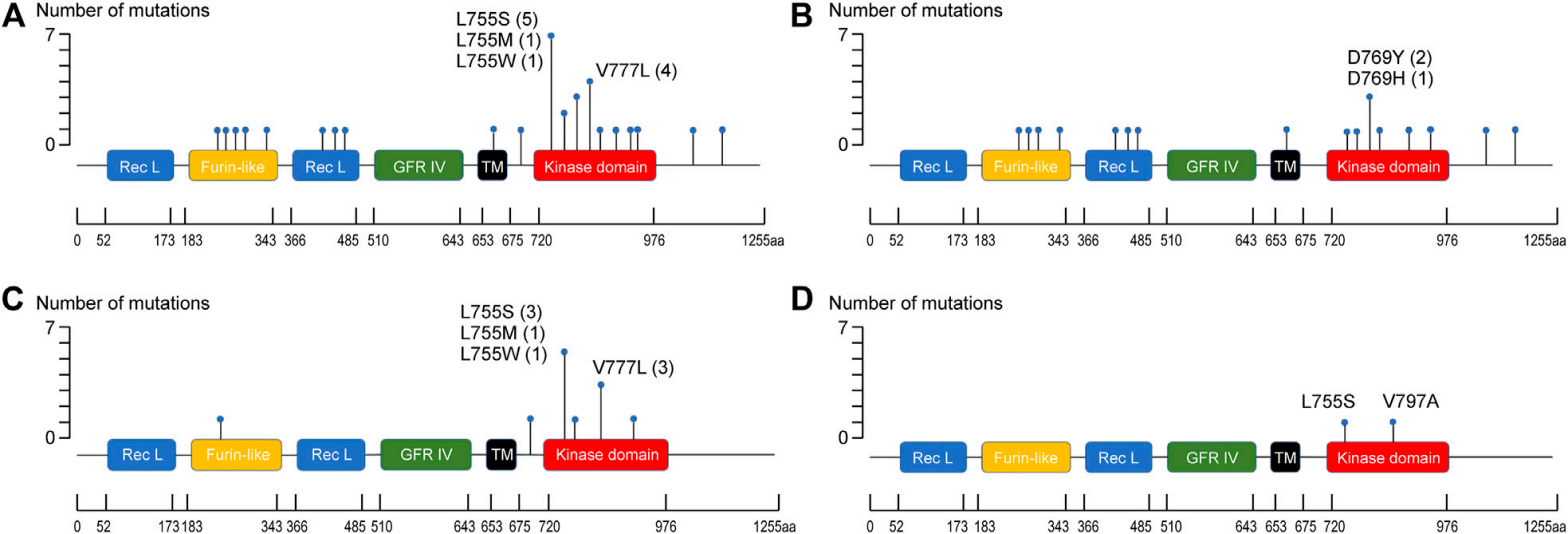
Distribution of *ERBB2* mutation sites. **(A)** Distribution of ERBB2 mutation sites in ERBB2-mutated BC. **(B)** Distribution of ERBB2 mutation sites in ERBB2-mutated IBC-NST. **(C)** Distribution of ERBB2 mutation sites in ERBB2-mutated c-ILC. **(D)** Distribution of ERBB2 mutation sites in ERBB2-mutated *p*-ILC.

The *ERBB2* mutations were associated with 32 types of amino acid changes in the following protein domains/regions: kinase domain (20/32, 62.5%), receptor-L domain (3/32, 9.4%), furin-like cysteine-rich domain (5/32, 15.6%), C-terminal region (2/32, 6.25%), the interdomain region (1/32, 3.1%), and the transmembrane region (1/32, 3.1%) (Figure 3). Substitutions at L755 (L755S, L755M, and L755W) and V777 (V777L) accounted for 34.4% of all amino acid mutations (11/32), especially in patients with c-ILC (66.7%) and *p*-ILC (50%) (Table 3). In contrast, in IBC-NST, the aforementioned L755 and V777L mutations accounted for approximately 11.2% (2/18) of all mutations (Table 3).

**TABLE 3:** The proportion of L755 and V777L in *ERBB2*.

	*ERBB2*-mutated BC (*n* = 32)		IBC-NST (*n* = 18)		c-ILC (*n* = 12)		*p*-ILC (*n* = 2)	
*ERBB2* mutation site	Number	%	Number	%	Number	%	Number	%
L755 (L755S, L755M, L755W)	7	21.9	1	5.6	5	41.7	1	50
V777L	4	12.5	1	5.6	3	25	0	0
Other sites	21	65.6	16	88.9	4	33.3	1	50

*ERBB2*-mutated BC: *ERBB2*-mutated breast cancer; IBC-NST: Invasive breast cancer-no special type; c-ILC: Classic invasive lobular carcinoma; *p*-ILC: Pleomorphic invasive lobular carcinoma.

### Comparison Between the Level of Immune Cell Infiltration in ERBB2-Mutated and Non-mutated BC

**FIGURE 4:**
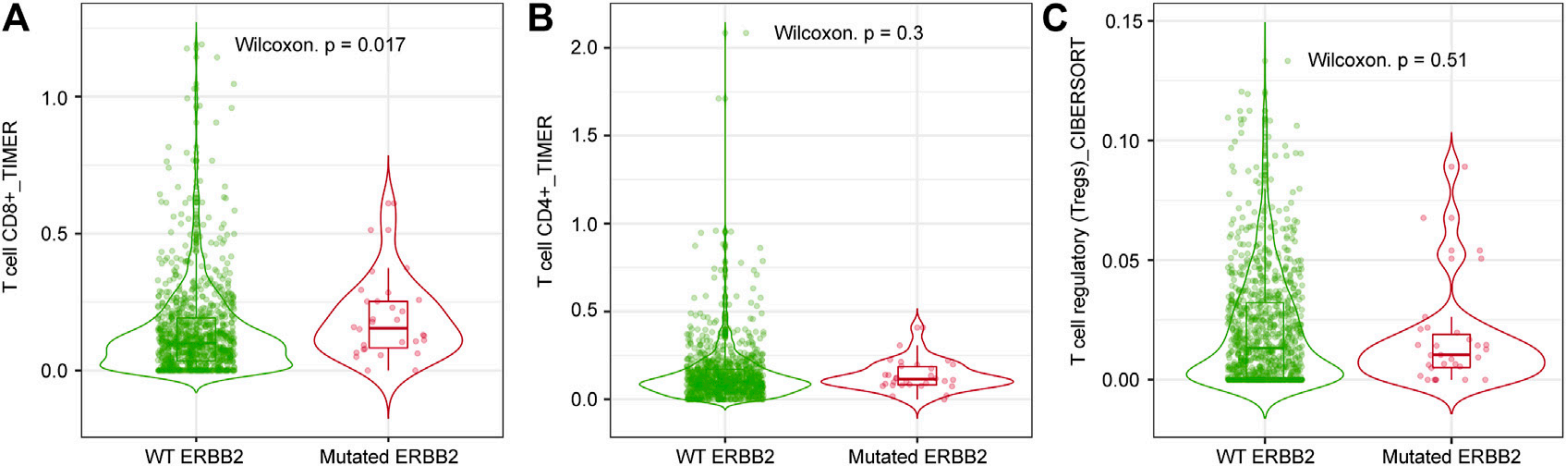
Comparison between the level of immune infiltration levels in *ERBB2*-mutated and non-mutated BC via TIMER2.0 analysis. **(A)** CD8^+^ T cell infiltration level in *ERBB2*-mutated and non-mutated BC (TIMER analysis). **(B)** CD4^+^ T cell infiltration level in *ERBB2*-mutated and non-mutated BC (TIMER analysis). **(C)** Treg infiltration level in *ERBB2*-mutated and non-mutated BC (CIBERSORT analysis).

CD8^+^ T cell infiltration (Figure 4A) was significantly higher in *ERBB2*-mutated BC than in their non-mutant counterparts (*p* < 0.05). In contrast, no differences in CD4^+^ T cell and Treg infiltration were observed between *ERBB2* mutated and non-mutated BC (Figures 4B,C), as determined by TIMER and CIBERSORT analyses using TIMER2.0.

### TMB and TILs in *ERBB2*-Mutated BC

**FIGURE 5:**
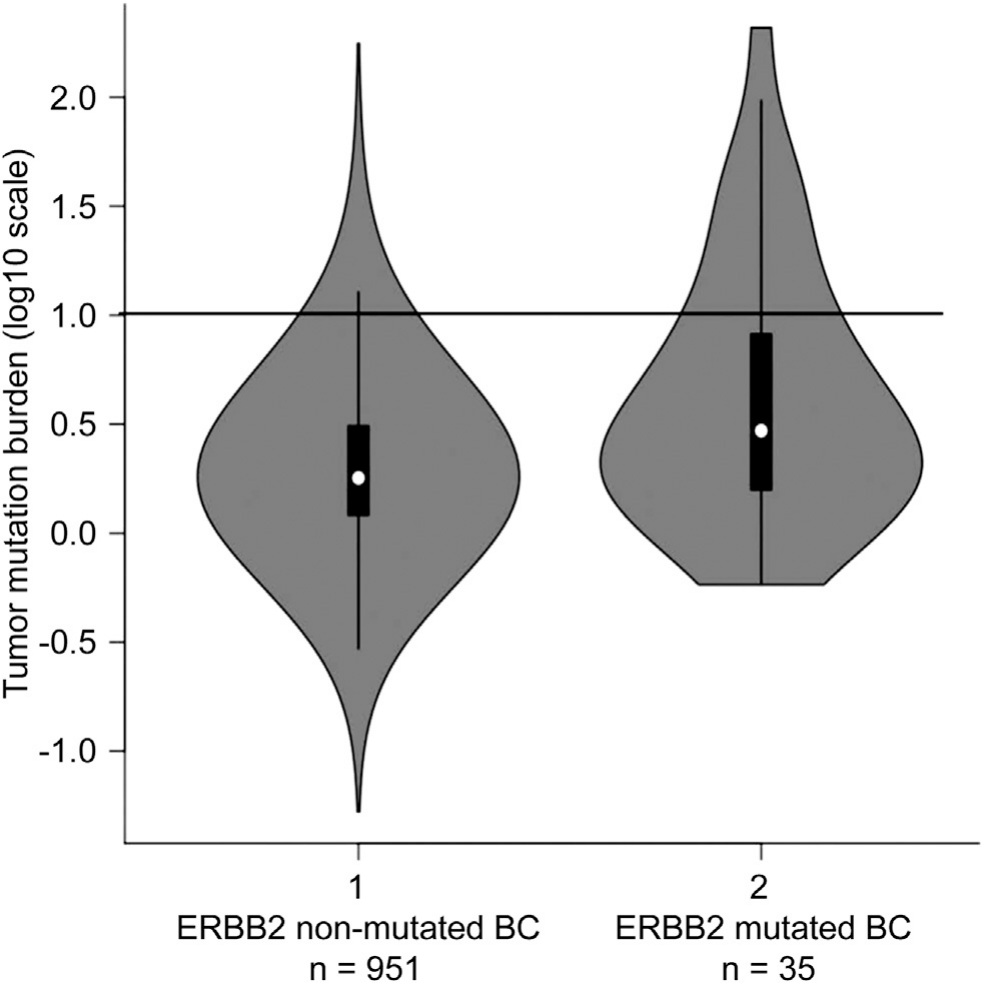
Tumour mutational burden (TMB) in *ERBB2* non-mutated BC (*n* = 951) and *ERBB2* mutated BC (*n* = 35) The black line indicating 10 mutations/megabase represents the threshold for TMB-high. For *ERBB2* non-mutated BC, the frequency of TMB-high was 3.0% (29/951); for *ERBB2*-mutated BC, the frequency was 22.9% (8/35).

**FIGURE 6:**
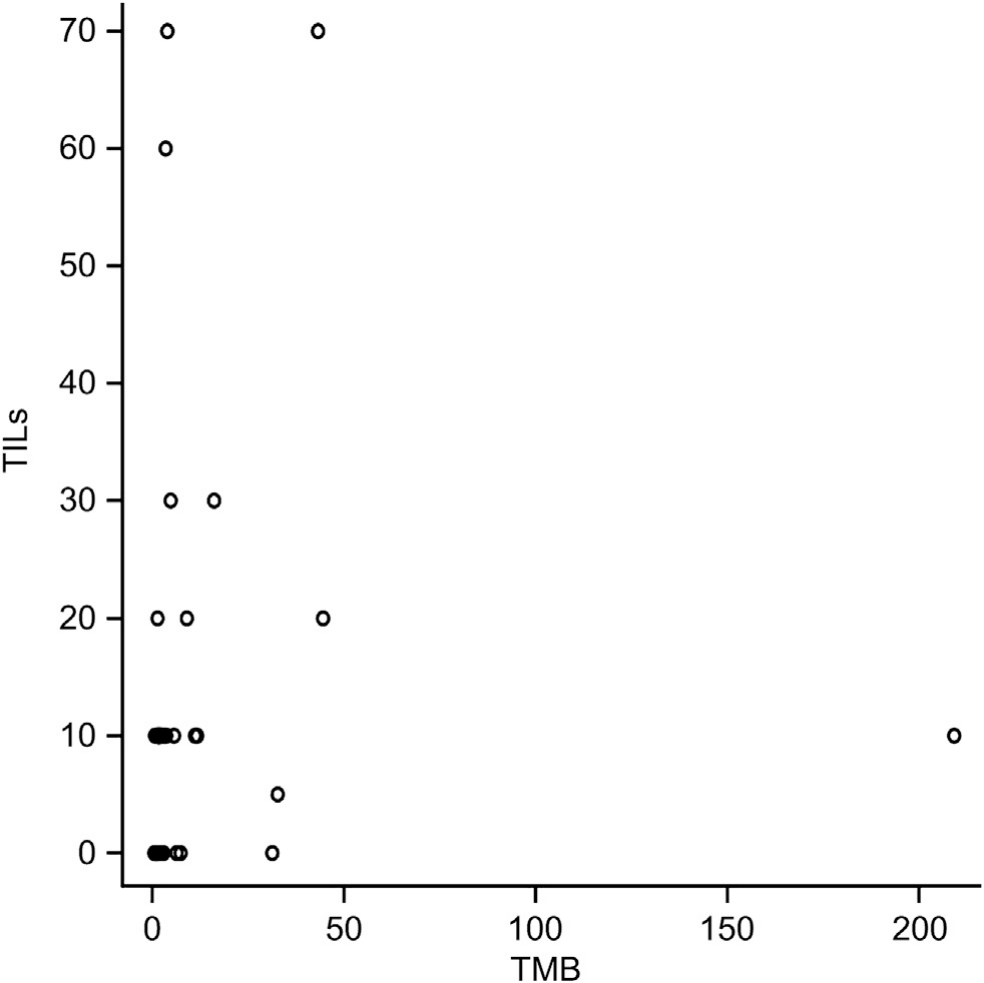
Correlation between tumor mutational burden (TMB) and tumour-infiltrating lymphocytes (TILs). Spearman’s rank correlation coefficient, 0.38 (*p* < 0.05).

**FIGURE 7:**
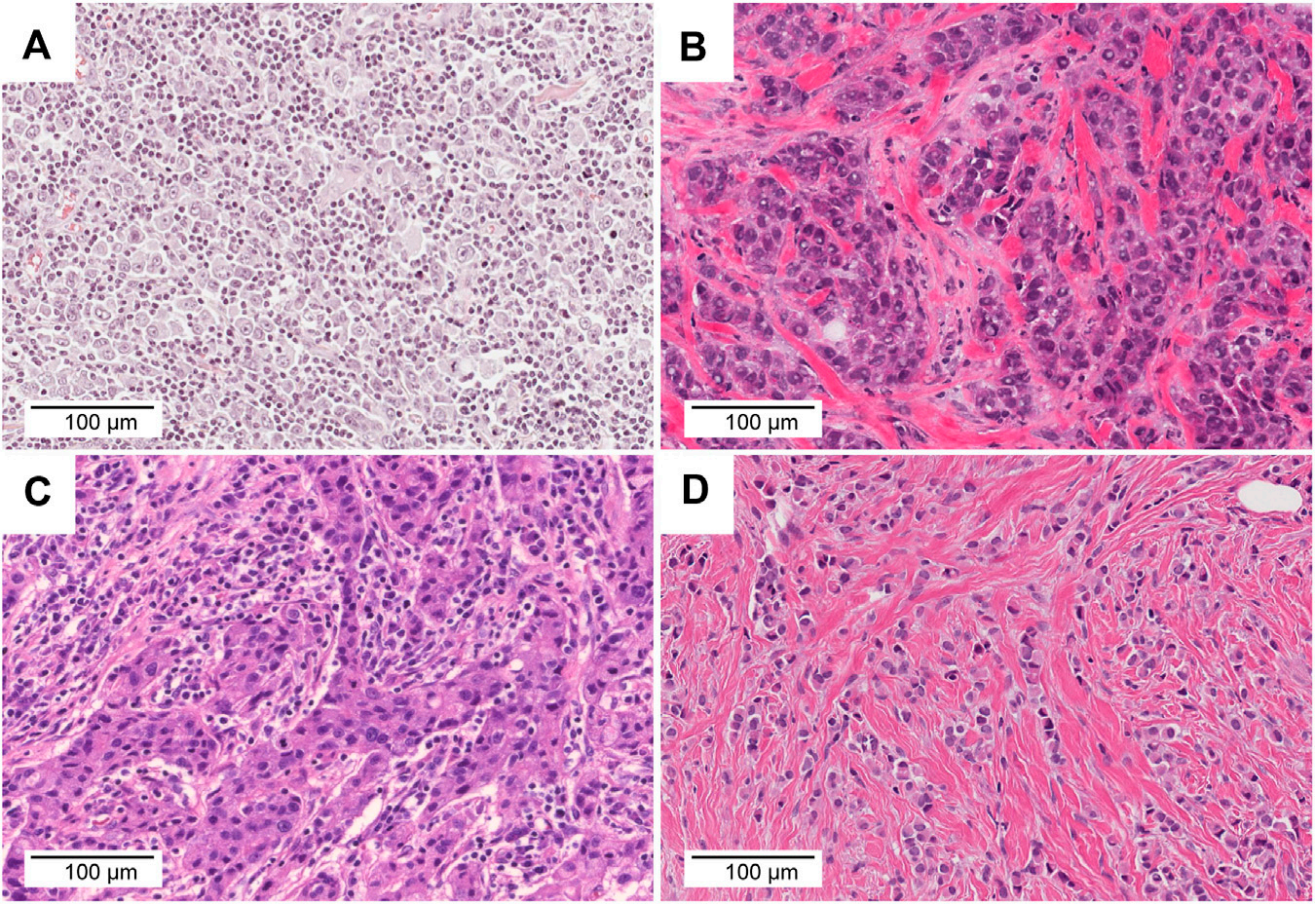
Comparison of TMB, TILs, and histology. **(A)** TCGA-AO-A128-01A. This tumour was classified as TMB-high and TIL-high (TMB = 43.3 mut/Mb, TILs = 70%). **(B)** TCGA-AN-A46-01A. This tumor was classified as TMB-high and TIL-low (TMB = 209.2 mut/Mb, TILs = 10%). **(C)** TCGA-OL-A66P-01A. This tumor was classified as TMB-low and TIL-high (TMB = 4.0 mut/Mb, TILs = 70%). **(D)** TCGA-AC-A3YI-01A. This tumor was classified as TMB-low and TIL-low (TMB = 0.6 mut/Mb, TILs = 0%). Scale bar = 100 μm, ×20 magnification.

The mean TMB in *ERBB2*-mutant BC was 13.6 mut/Mb (median = 3.0 mut/Mb), while the mean TMB in the TCGA-BRCA cohort without *ERBB2* mutations (*n* = 951) was 3.2 mut/Mb (median = 1.8 mut/Mb). The TMB in *ERBB2*-mutated BC was significantly higher than that *ERBB2* non-mutated BC (*p* < 0.01) (Table 2; Figure 5). Furthermore, 3.0% (29/951) of the samples in the ERBB2 non-mutated BC were classified as TMB-high based on the calculated cut-off of 10 mut/Mb. In comparison, 22.9% (8/35) of the *ERBB2*-mutated BC samples were TMB-high. Furthermore, 8.6% (3/35) of *ERBB2*-mutated BC samples were TILs-high, 14.3% (5/35) were TIL-intermediate, and 77.1% (27/35) were TILs-low, including 12 samples with no TILs (34.3%). The correlation coefficient for the relationship between TMB and TILs was 0.38 (Spearman's rank correlation, *p* < 0.05), indicating a weak positive correlation (Figures 6, 7).

## Discussion

We characterized the clinicopathological features, immune infiltration levels, TMB (mut/Mb), and TILs (%) associated with *ERBB2*-mutated BC using bioinformatics. *ERBB2*-mutated BC was identified in 3.5% of TCGA-BRCA samples, concurrent with a previous report [3]. In this study, among the 35 samples, 60% were IBC-NST and 40% were ILC (c-ILC, *p*-ILC). As ILC typically accounts for 5–15% of all BCs [15], ILC appears to be enriched in *ERBB2* mutations. A similar trend has been reported previously [4, 7]. In *ERBB2*-mutated BC, ILC is frequently related to a high frequency of *CDH1* mutations. In this study, 40% (14/35) of the *ERBB2* mutant cases harbored *CDH1* mutation.

In our study, missense mutations in the kinase domain were the most common *ERBB2* mutation type in BC. The mutation type and site differ among tumor types. For example, *ERBB2* mutations in micropapillary urothelial carcinoma of the urinary bladder are predominantly present in the extracellular domain [23].

In this study, L755 mutations and V777L accounted for 34.4% of all *ERBB2* mutations in BC. Remarkably, more than half of c-ILC and *p*-ILC samples revealed the presence of amino acid change at L755 and V777L. A correlation has been reported between *p*-ILC and amino acid change at L755 [14]; however, in this study, a similar correlation was also observed for c-ILC. Together with previous reports [14, 24], our study shows that kinase domain (predominantly L755 site and V777L substation, especially in lobular carcinoma) is a hot spot for *ERBB2* mutation in BC. A previous study integrated three cohorts (METABRIC, TCGA, MSK-IMPACT) and calculated the frequency of the *ERBB2* mutation (2.2%, 34/1,580). The *ERBB2* mutation was enriched in ILC with a prevalence of 5.7% (*n* = 16) vs. 1.4% in IDC (*n* = 18) [25]. Based on these findings, it is thought that an analysis of other data sets (METABRIC and MSK-IMPACT) will yield the same results.

Previous studies have reported that irreversible tyrosine kinase inhibitors (TKIs, such as neratinib and afatinib) are useful for treating *ERBB2*-mutated BC [1, 26, 27]. Ongoing clinical trials are investigating the effect of neratinib (SUMMIT trial; NCT01953926) and afatinib (NCI-MATCH; NCT02465060) in patients with *ERBB2*-mutant cancers. However, neratinib-resistant BC has been previously reported [26]. Acquired tolerance to TKIs occurs via multiple mechanisms, including gatekeeper mutations and “bypass” resistance [28]. The acquisition of therapeutic resistance in cancer cells is a major challenge of molecular targeted therapy. Therefore, we evaluated TMB and TILs to investigate the possibility of immunotherapy as an alternative treatment, including the use of TKIs, such as neratinib and afatinib.

TMB is a predictive biomarker for the response to immune checkpoint inhibitor (ICI) therapy, and some clinical studies have reported a response to immunotherapy based on a high TMB [29, 30]. In fact, in June 2020, the US-FDA approved pembrolizumab for treating solid tumors in adults and children with unresectable or metastatic high TMB (≥10 mut/Mb) solid tumors based on the results of the KEYNOTE-158 trial [31]. In the present study, we found that the TMB is significantly higher in *ERBB2*-mutated BC than in *ERBB2* non-mutated BC samples without *ERBB2* mutations (*p* < 0.01). In BC, the TMB is usually lower (2.6 mut/Mb) than in other carcinomas, such as lung cancer [21, 22]. In a previous study based on 3,969 primary and metastatic BC samples, approximately 5% of the samples were classified as TMB-high [22]. However, in this study, 22.9% of the *ERBB2*-mutated BC samples were TMB-high, and *ERBB2*-mutated BC was regarded as a subset of TMB-high BC. TILs are a favorable prognostic factor and are associated with the response to PD-1/PD-L1 inhibitors in BC [32]. The samples used in our study exhibited the following TILs frequencies: high (8.6%; 3/35); intermediate (14.3%; 5/35), and low (77.1%; 27/35), including 12 samples with no TILs (34.3%). In a previous study, 44.2% of the samples were TILs-low, 36.3% were TILs-intermediate, and 19.2% were TIL-high [33]. In another study, TILs were lacking in 16% of the samples [34]. In our study, *ERBB2*-mutated BC was characterized by low TILs count. TILs are reportedly higher in triple-negative and Her2-positive BC subtypes than in the luminal subtype. Moreover, ILCs are TILs-low compared with other histological types [33]. The TILs pattern in *ERBB2*-mutated BC resembled the pattern observed in hormone receptor-positive/Her2-negative BC and ILC [34]. Although *ERBB2*-mutated BCs are classified as TMB-high and TILs-low, a positive correlation was observed between TMB and TILs in this study. TIMER2.0 analysis revealed that the CD8^+^ T infiltration level of *ERBB2*-mutated BC was significantly higher than that of *ERBB2* non-mutated BC. In *ERBB2*-mutated BC, the CD8 + T cell infiltration level was considered to be up-regulated, and subpopulations of TILs may contain much CD8^+^ T cells. However, further investigations are needed to confirm this.

This study demonstrated that 22.9% of the *ERBB2*-mutated BCs were TMB-high BC; in contrast, 77.1% of them were TILs-low. From this result, it is questionable whether *ERBB2*-mutated cases would really benefit from ICI therapy. Further research is needed to determine whether it is an indication for ICI therapy. Since TMB estimation are highly variable [35], with slower turn-around times and high costs, further studies are required to validate the correlations with between other biomarkers, such as PD-L1 immunostaining and microsatellite instability (MSI), and TMB. A recent study reported that a machine learning algorithm, Image2TMB, can predict the TMB directly from images of H&E-stained histopathological sections [36]; however, this method is still not used in the clinical setting, although it may be a novel method of estimating the TMB easily.

This study has some limitations. This result was obtained from one dataset (TCGA-BRCA), and it is necessary to verify the obtained result by laboratory research in the future.

In conclusion, The TMB and CD8^+^ T cell infiltration level in *ERBB2*-mutated BC samples was significantly higher than that in *ERBB2* non-mutated BC. Additionally, 22.9% of the *ERBB2*-mutated BC samples were identified as TMB-high, and a positive correlation was identified between TMB and TILs.

## Data Availability

The original contributions presented in the study are included in the article/Supplementary Material, further inquiries can be directed to the corresponding author.
